# Psychosocial Correlates of Exclusive and Dual Nicotine Use among Adolescents in a Context of E-Cigarette Sales Prohibition

**DOI:** 10.1007/s10900-026-01632-4

**Published:** 2026-08-31

**Authors:** Barbara de Freitas Quitério, Patricia Paiva de Oliveira Galvão, Sheila Cavalcante Caetano, Zila M. Sanchez, Juliana Y. Valente

**Affiliations:** 1 Department of Preventive Medicine, Universidade Federal de São Paulo (UNIFESP), Rua Botucatu, 740 – Vila Clementino, São Paulo, SP 04023-062 Brazil; 2 Department of Psychiatry, Universidade Federal de São Paulo (UNIFESP), Rua Borges Lagoa, 570 – Vila Clementino, São Paulo, SP 04038-000 Brazil

**Keywords:** Electronic cigarettes, Conventional cigarettes, Adolescents, Nicotine, Psychosocial factors, Health beliefs

## Abstract

Although the sale of electronic cigarettes (ECs) has been prohibited in Brazil since 2009, experimentation among adolescents has increased substantially in recent years. Understanding the psychosocial factors associated with different nicotine use patterns in this regulatory context may help inform more effective prevention strategies. This study examined sociodemographic characteristics and psychosocial factors associated with exclusive EC use, exclusive conventional cigarette use, and dual use among Brazilian adolescents. A cross-sectional school-based survey was conducted with 2,939 adolescents from public schools in São Paulo State, Brazil. Participants completed an anonymous self-administered questionnaire. Multilevel multinomial logistic regression models were used to examine factors associated with exclusive EC use, exclusive conventional cigarette use, dual use, and non-use. The lifetime prevalence of exclusive EC use, exclusive conventional cigarette use, and dual use was 13.3%, 2.9%, and 12.9%, respectively. Girls were more likely to report exclusive EC use (RRR = 1.48; 95% CI: 1.16–1.88) and dual use (RRR = 1.52; 95% CI: 1.20–1.93), whereas boys were more likely to report exclusive conventional cigarette use. Perceived peer use and ease of access were strongly associated with both exclusive EC use and dual use, with consistently stronger associations among dual users. Adolescents who believed that ECs help reduce cigarette smoking were more likely to report exclusive EC use and dual use, whereas perceiving ECs as highly addictive was associated with lower odds of both exclusive EC use (RRR = 0.49; 95% CI: 0.35–0.67) and dual use (RRR = 0.44; 95% CI: 0.25–0.77). Psychosocial factors were differentially associated with nicotine use patterns among adolescents living in a context where EC sales are prohibited. These findings have important implications for community- and school-based prevention initiatives by highlighting the need to address perceived accessibility, peer norms, and misconceptions about electronic cigarettes in addition to regulatory measures.

## Introduction

According to the World Health Organization (WHO), the tobacco epidemic is the leading cause of death, illness, and impoverishment worldwide, accounting for over 8 million deaths annually [1]. In recent years, there has been a shift in nicotine consumption patterns. While the use of manufactured cigarettes has declined, the number of users of electronic cigarettes (ECs) and related products has rapidly increased, especially among young people. This rise is often attributed to appealing flavors, sleek designs, and aggressive marketing on social media platforms [2]. Although ECs have been promoted as a harm reduction strategy for smokers [3, 4], they are not risk-free. Studies have found that ECs may contain higher nicotine concentrations than conventional cigarettes [5, 6], as well as toxic and carcinogenic substances—such as formaldehyde and acetaldehyde—that can adversely affect the cardiovascular and pulmonary systems and pose a risk of physical injury [7, 8].

Adolescents are particularly vulnerable to the harmful effects of compounds present in ECs, which may negatively affect brain development [9]. The combined use of ECs and conventional cigarettes may further amplify these risks. Evidence suggests that adolescents who have used ECs are at higher risk of initiating cigarette smoking [10–12]. Furthermore, dual use—defined as the concurrent use of ECs and conventional cigarettes—is of particular concern, as it has been associated with higher levels of nicotine dependence and increased health risks [13, 14].

In Brazil, the importation and sale of ECs are strictly prohibited. While conventional cigarette experimentation continued to decline among Brazilian adolescents, decreasing from 22.6% in 2019 to 18.5% in 2024, lifetime electronic cigarette use increased sharply over the same period, rising from 16.8% to 29.6%. Electronic cigarette experimentation was more common among girls (31.7%) than boys (27.4%), highlighting a substantial shift in nicotine use patterns among adolescents [15]. Despite these findings, national estimates on dual use among Brazilian adolescents remain unavailable, revealing a critical gap in understanding this emerging pattern.

To prevent nicotine use among adolescents, it is essential to understand the factors associated with different nicotine consumption patterns—such as exclusive EC use, exclusive cigarette use, and dual use. International evidence suggests that psychosocial factors related to health harms and the addictive potential of nicotine products strongly influence adolescents’ decisions to try and continue using them. A systematic review conducted in countries such as the United States, United Kingdom, and Australia found that at least one-third of adolescents believe ECs are less harmful and less addictive than conventional cigarettes [16]. Similarly, a study in Australia reported that nearly half of adolescents perceived ECs as helpful in quitting or reducing traditional cigarette use [17].

In Latin America, studies conducted in countries such as Mexico, Colombia, and Argentina have revealed widespread misinformation about EC risks, with up to 86% of adolescents unaware of their harms and up to 43% believing they are less harmful [18–20]. These findings are concerning, as higher risk perceptions about nicotine products are linked to lower odds of use, including dual use [21]. A recent review of EC use in Latin America emphasized that adolescents who perceive ECs as less harmful are more likely to initiate use; however, no studies from Brazil were included in that review, further illustrating the scarcity of evidence in the region [12].

In Brazil, few studies have examined psychosocial factors associated with adolescent e-cigarette use, particularly harm perception, perceived addictiveness, and peer norms. Moreover, little is known about whether these factors differ across distinct nicotine use patterns, especially dual use, in a setting where electronic cigarette sales have been prohibited for more than a decade despite the marked increase in adolescent experimentation. Understanding these associations may provide insights into different patterns of adolescent nicotine use within a restrictive regulatory environment and help inform prevention strategies. Therefore, this study examined sociodemographic characteristics and psychosocial factors associated with lifetime exclusive e-cigarette use, exclusive conventional cigarette use, and dual use among Brazilian adolescents.

### Methods

This cross-sectional study was conducted with 2,939 adolescents from three small municipalities in the state of São Paulo, Brazil (Iracemápolis, Salesópolis, and Biritiba Mirim). Data collection took place between August and December 2024 in public schools, using an anonymous, self-administered questionnaire applied in classrooms to students from the 7th grade of lower secondary school to the final year of upper secondary school. All schools (*n* = 9) in the participating municipalities were invited to join the study, and all students enrolled in the eligible grades of participating schools were included. A census approach was adopted for these grades, and no sample size calculation was performed.

Parents or legal guardians provided written informed consent prior to data collection, and adolescents signed an assent form on the day of participation. All study procedures were approved by the Research Ethics Committee of the University and were conducted in accordance with the ethical standards of the Declaration of Helsinki (1964) and its later amendments.

### Instruments and Variables

Data were collected using an anonymous, self-administered questionnaire, completed by students on smartphones provided by the research team in the classroom, without the presence of the teacher. All students from the 7th grade through the final year of high school in participating schools completed the questionnaire.

The outcome variable was constructed from two dichotomous questions: “Have you ever tried vaping/pod/ECs?” and “Have you ever tried conventional cigarettes?”, with “yes” or “no” response options. Based on the combination of answers, a mutually exclusive categorical variable was created with four groups: (1) never used either product, (2) exclusive use of conventional cigarettes, (3) exclusive use of ECs, and (4) dual use (experience with both products).

Perceptions about ECs as a smoking cessation strategy were assessed with the question: “Do you believe that ECs can help smokers reduce or quit traditional cigarette use?”, with “yes” and “no” response options.

Harm perception related to EC use were measured through the question: “How harmful do you think e-cigarettes are to health?”, with four response options ranging from “not harmful at all” to “extremely harmful.”

Perceived addictiveness of ECs was evaluated using two questions: “How addictive do you think ECs are?” and “Compared to conventional tobacco cigarettes, how addictive do you think ECs are?”, with responses ranging from “not at all addictive” to “extremely addictive.”

Normative beliefs were assessed using the question: “How many of your peers use vaping/pod/ECs?”, with response options: “none or less than half,” “about half,” and “more than half or all.”

Perceived availability of ECs was measured by the question: “How easy would it be for you to buy a vape/pod/ECs?”, with three response options: “easy,” “moderate,” and “difficult.”

Sociodemographic variables included gender, age, and socioeconomic status, the latter assessed using the Brazilian Economic Classification Criteria (ABEP, 2022), which considers ownership of household goods, access to services, and the education level of the household head. The composite score ranges from 0 to 100 and classifies individuals into four socioeconomic groups: A (45–100 = high), B (29–44 = upper-middle), C (17–28 = lower-middle), and D/E (0–16 = low).

### Data Analysis

Initially, all study variables were analyzed descriptively. Categorical variables were summarized using absolute frequencies and prevalence rates, while continuous variables were summarized using means and standard deviations. Subsequently, both univariate and multivariate multilevel multinomial logistic regression models were used to examine associations between sociodemographic characteristics, psychosocial factors, and three lifetime use patterns: exclusive use of electronic cigarettes, exclusive use of conventional cigarettes, and dual use. Adolescents who reported no lifetime use were used as the reference group in all models.

Given the hierarchical structure of the data (students nested within schools), robust standard errors were estimated by adjusting for school-level clustering using the vce (cluster) option. All analyses were performed using Stata 17.0 software.

## Results

Table 1 presents the sample characteristics, which included 2,939 adolescents with a mean age of 14.63 years. There was a slight predominance of males in the sample (50.9%), and the majority belonged to socioeconomic class B (45.8%), followed by class C (36.1%). Among the participants, 13.3% reported exclusive use of EC, 2.9% reported exclusive use of conventional cigarettes, and 12.9% reported dual use of both products, while 70.9% reported never having used either.

**Table 1:** Description of sociodemographic variables according to lifetime use of electronic cigarettes (ECs) and conventional cigarettes (*n* = 2,939)

	Total *n* = 2,939		Never Use *n* = 2,085 (70,9%)		Only ECs *n* = 391 (13,3%)		Only conventional cigarettes *n* = 85 (2,9%)		Dual Use *n* = 378 (12,9%)					P-valor
	*N*	%	*N*	%	*N*	%	*N*	%	*N*			%	*P*-valor	
*Gender*														< 0.001
Boys	1485	51,0	1099	53,2	172	44,1	53	63,9	161		42,9			
Girls	1429	49,0	967	46,8	218	55,9	30	36,1	214		57,1			
Age (mean +/-)	14,6 +/-1,7		14,4 +/-1,7		15,0 +/- 1,7		14,8 +/- 1,7		15,3 +/- 1,7					< 0.001
*Socioeconomic Status*														0.034
A	369	12,6	239	11,5	56	14,3	10	11,8	64		16,9			
B	1346	45,8	959	46,0	191	48,9	37	43,5	159		42,1			
C	1063	36,2	770	36,9	131	33,5	30	35,3	132		34,9			
D/E	161	5,4	117	5,6	13	3,3	8	9,4	23		6,1			

Socioeconomic Status based on the Brazilian Economic Classification Criteria - ABEP.

Among students who reported exclusive use of ECs, most were female (55.9%), a pattern also observed among dual users (57.0%). Conversely, among those who reported exclusive use of conventional cigarettes, the majority were male (63.9%). The mean age was lowest among those who had never tried either product (14.4 years) and highest among dual users (15.3 years). Regarding socioeconomic class, all patterns of nicotine users were predominantly from class B.

In terms of perceptions and beliefs regarding ECs, most adolescents believed that EC use was widespread among their peers: 41.5% stated that more than half or all of their friends used the product. Additionally, 33.4% considered ECs to be easily accessible. Regarding risk perception, 76.1% of adolescents believed that ECs were very or extremely harmful to health, and 75.6% classified them as extremely addictive. Similarly, 81.1% believed that ECs are as addictive or more addictive than conventional cigarettes (Table 2).

**Table 2:** Description of explanatory variables according to lifetime use of electronic cigarettes (ECs) and conventional cigarettes (*n* = 2,939)

	Total *n* = 2,939		Never Use *n* = 2,085 (70,9%)		Only ECs *n* = 391 (13,3%)		Only conventional cigarettes *n* = 85 (2,9%)		Dual Use *n* = 378 (12,9%)		*p*-valor
	*N*	%	*N*	%	*N*	%	*N*	%	*N*	%	
*Normative Beliefs*											< 0.001
None or less than half	1006	34,2	843	40,4	74	18,9	31	36,5	58	15,3	
About half use	714	24,3	509	24,4	109	27,9	17	20,0	79	20,9	
More than half or all use	1219	41,5	733	35,2	208	53,2	37	43,5	241	63,8	
*Perceived Availability*											< 0.001
Difficult	1346	45,8	1152	55,3	95	24,3	38	44,7	61	16,1	
Moderate	612	20,8	392	18,7	107	27,4	21	24,7	92	24,3	
Easy	981	33,4	541	26,0	189	48,3	26	30,6	225	59,6	
*Harm Perception*											< 0.001
Not or slightly harmful	307	10,5	193	9,3	50	12,8	14	16,5	50	13,2	
Moderately harmful	395	13,4	195	9,3	93	23,8	12	14,1	95	25,1	
Extremely harmful	2237	76,1	1,69	81,4	248	63,4	59	69,4	233	61,7	
*Perceived Addictiveness of EC*											< 0.001
Not at all/slightly addictive	320	10,9	187	9,0	60	15,3	13	15,3	60	15,9	
Moderately addictive	397	13,5	209	10,0	92	23,5	13	15,3	83	22,0	
Very or extremely addictive	1,22	75,6	1689	81,0	239	61,2	59	69,4	235	62,2	
*Perceived Addictiveness of E-Cigarettes in relation to conventional cigarettes*											< 0.001
Less addictive	550	18,7	316	15,2	115	29,4	20	23,5	99	26,2	
Equally addictive/more addictive	2,89	81,3	1769	84,8	276	70,6	65	76,5	279	73,8	
*Belief that e-cigarettes reduce smoking*											< 0.001
No	2120	72,1	1609	77,2	236	60,4	61	71,8	214	56,6	
Yes	819	27,9	476	22,8	155	39,6	24	28,2	164	43,4	

Normative beliefs: adolescents’ perception of e-cigarette use among their peers.

Belief that e-cigarettes reduce smoking: the belief that e-cigarettes can help reduce or stop the use of conventional cigarettes.

Univariate analysis results are presented in Table 3, showing associations between sociodemographic characteristics, perceptions, beliefs, and the three lifetime use patterns, using non-use as the reference group. A gradient was observed for normative beliefs: the greater the perception that peers used ECs, the greater the likelihood of use. Compared to adolescents who believed that “none or less than half” of their peers used ECs, those who believed that “more than half or all” of their peers used ECs, had increased odds of exclusive EC use (RRR = 2.55; 95% CI: 1.65–3.95), exclusive conventional cigarette use (RRR = 1.49; 95% CI: 1.03–2.13), and dual use (RRR = 3.27; 95% CI: 2.19–4.90).

**Table 3:** Factors associated with lifetime use of electronic cigarettes (ECs) and conventional cigarettes – Multivariate analyses (*n* = 2,914)

	Only ECs			Only conventional cigarettes			Dual use		*p*-valor
	RRR	IC 95%	*p*-valor	RRR	IC 95%	*p*-valor	RRR	IC 95%	
Normative Beliefs									
None or less than half	REF								
About half use	2.43	1.85; 3.21	< 0.001	0.90	0.61; 1.34	0.632	2.25	1.54;3.30	< 0.001
More than half or all use	3.23	2.18; 4.8	< 0.001	1.37	0.94; 2.00	0.09	4.77	3.55; 6.45	< 0.001
Perceived Availability									
Difficult	REF								
Moderate	3.30	2.24; 4.90	< 0.001	1.62	1.07; 2.45	0.021	4.43	3.13; 6.26	< 0.001
Easy	4.23	3.04; 5.90	< 0.001	1.45	0.90; 2.34	0.120	7.85	5.25;11.75	< 0.001
Harm Perception									
Not or slightly harmful	REF								
Moderately harmful	1.84	1.22; 2.76	0.003	0.84	0.42; 1.72	0.648	1.88	1.27; 0.79	0.002
Extremely harmful	0.56	0.36; 0.87	0.010	0.48	0.28; 0.80	0.006	0.53	0.35; 0.80	0.002
Perceived Addictiveness of EC									
Not at all or slightly addictive	REF								
Moderately addictive	1.37	0.99; 1.90	0.054	0.89	0.50	0.707	1.23	0.98; 1.55	0.067
Very or extremely addictive	0.44	0.30; 0.64	< 0.001	0.50	0.36	< 0.001	0.43	0.32; 0.58	< 0.001
Perceived Addictiveness of E-Cigarettes in relation to conventional cigarettes									
Less addictive	REF								
Equally addictive/more addictive	0.42	0.29; 0.62	< 0.001	0.58	0.40; 0.83	0.004	0.50	0.41;0.60	< 0.001
Belief that e-cigarettes reduce smoking									
No	REF								
Yes	2.22	1.89; 2.60	< 0.001	1.32	0.83; 2.12	0.232	2.60	2.02; 3.32	< 0.001
Gender									
Boys	REF								
Girls	1.44	1.13; 1.83	0.003	0.64	0.45; 0.90	0.012	1.51	1.15; 1.98	0.002
Age (mean +/-)	1.20	1.13; 1.28	< 0.001	1.14	0.97; 1.34	0.111	1.32	1.21; 1.43	< 0.001
Socioeconomic Status									
A	REF								
B	0.85	0.56; 1.29	0.445	0.92	0.51; 1.66	0.788	0.61	0.51; 0.75	< 0.001
C	0.72	0.49; 1.06	0.098	0.93	0.37; 2.30	0.877	0.64	0.42; 0.96	0.030
D/E	0.47	0.27; 0.80	0.006	1.63	0.81; 3.29	0.168	0.73	0.46; 1.15	0.183

Normative beliefs: adolescents’ perception of e-cigarette use among their peers.

E-cigarettes reduce conventional smoking: the belief that e-cigarettes can help reduce or stop the use of conventional tobacco cigarettes.

A similar pattern was found for perceived availability: the easier the perceived access, the greater the likelihood of use. Compared to those who reported “difficult” access, adolescents reporting “moderate” or “easy” access had higher odds of exclusive EC use (RRR = 2.62; 95% CI: 1.71–4.00 and RRR = 2.85; 95% CI: 2.01–4.04, respectively) and dual use (RRR = 3.30; 95% CI: 2.50–4.35 and RRR = 4.72; 95% CI: 3.20–6.96). Moderate access was also associated with exclusive use of conventional cigarettes (RRR = 1.63; 95% CI: 1.06–2.50) (Table 3). 

Regarding harm perception, in the univariate analysis, adolescents who perceived ECs as moderately harmful had higher odds of exclusive EC use (RRR = 1.84; 95% CI: 1.22–2.76) and dual use (RRR = 1.88; 95% CI: 1.27–2.79), compared to those who considered ECs “not or slightly harmful.” Conversely, perceiving ECs as very or extremely harmful was associated with lower odds of exclusive EC use (RRR = 0.56; 95% CI: 0.36–0.87) and dual use (RRR = 0.53; 95% CI: 0.36–0.76) (Table 4). However, in the multivariate model, only the perception that ECs are moderately harmful remained statistically significant, with increased odds of exclusive EC use (RRR = 1.49; 95% CI: 1.00–2.20) (Table 3). These findings suggest that perceiving e-cigarettes as moderately harmful does not serve as a protective factor against use; only the perception of high harm appears to be associated with lower odds of engagement.

**Table 4:** Factors associated with lifetime use of electronic cigarettes (ECs) and conventional cigarettes – Univariate analyses (*n* = 2,939)

	Only ECs			Only conventional cigarettes			Dual use		*p*-valor
	RRR	IC 95%	*p*-valor	RRR	IC 95%	*p*-valor	RRR	IC 95%	
*Normative Beliefs*									
None or less than half	REF								
About half use	2.03	1.46; 2.82	< 0.001	0.83	0.47; 1.46	0.530	1.70	1.05; 2.75	0.030
*More than half or all use*	2.55	1.65; 3.95	< 0.001	1.49	1.03; 2.13	0.030	3.27	2.19; 4.90	< 0.001
Perceived Availability									
Difficult	REF								
Moderate	2.62	1.71; 4.00	< 0.001	1,63	1.06; 2.50	0.024	3.30	2.50; 4.35	< 0.001
Easy	2.85	2.01; 4.04	< 0.001	1,30	0.83; 2.02	0.251	4.72	3.20; 6.96	< 0.001
*Harm Perception*									
Not or slightly harmful	REF								
Moderately harmful	1.49	1.00; 2.20	0.049	0.91	0.52; 1.60	0.759	1.45	0.90; 2.35	0.125
Extremely harmful	0.83	0.55; 1.25	0.389	0.68	0.27, 1.71	0.414	0.72	0,37; 1,40	0.321
*Perceived Addictiveness of EC*									
Not at all or slightly addictive	REF								
Moderately addictive	1.05	0.75; 1.47	0.753	1.11	0.79; 1.58	0.550	0.86	0.59; 1.27	0.460
Very or extremely addictive	0.49	0.35; 0.67	< 0.001	0.74	0.39; 1.44	0.387	0.44	0.25; 0.77	0.004
*Perceived Addictiveness of E-Cigarettes in relation to conventional cigarettes*									
Less addictive	REF								
Equally addictive or more addictive	0.65	0.42; 1.01	0.058	0.81	0.48; 1.38	0.447	0.810	0.64; 1.02	0.078
*Belief that e-cigarettes reduce smoking*									
No	REF								
Yes	1.70	1.33; 2.18	< 0.001	1.19	0.71; 2.00	0.502	1.96	1.36; 2.82	< 0.001
*Gender*									
Boys	REF								
Girls	1,48	1.16; 1.88	0.001	0.64	0.47; 0.87	0.004	1.52	1.20; 1.93	0.001
Age (mean +/-)	1.14	1.05; 1.23	0.001	1.12	0.95; 1.32	0.165	1.23	1,13; 1.35	< 0.001
*Socioeconomic Status*									
A	REF								
B	0.94	0.61, 1.43	0.779	0.95	0.51; 1,74	0.865	0.70	0.55; 0.90	0.004
C	0.78	0.49; 1.24	0.296	0.94	0.36; 2.45	0.900	0.70	0.45; 1.10	0.131
D/E	0.52	0.30; 0.89	0.016	1.70	0.93; 3.04	0.081	0.81	0.47; 1.40	0.466

Normative beliefs: adolescents' perception of e-cigarette use among their peers.

E-cigarettes reduce conventional smoking: belief that e-cigarettes can help reduce or stop the use of conventional tobacco cigarettes.

Regarding perceived addictiveness, adolescents who classified ECs as very or extremely addictive had lower odds of exclusive EC use (RRR = 0.49; 95% CI: 0.35–0.67) and dual use (RRR = 0.44; 95% CI: 0.25–0.77), compared to those who considered them “not or slightly addictive.” Additionally, adolescents who believed that ECs help reduce traditional cigarette use had higher odds of exclusive EC use (RRR = 1.70; 95% CI: 1.33–2.18) and dual use (RRR = 1.96; 95% CI: 1.36–2.82) compared with those who did not hold this belief **(**Table 3). 

In terms of sociodemographic characteristics, girls were more likely to report exclusive EC use (RRR = 1.48; 95% CI: 1.16–1.88) and dual use (RRR = 1.52; 95% CI: 1.20–1.93), and less likely to report exclusive use of conventional cigarettes (RRR = 0.64; 95% CI: 0.47–0.87), compared to boys. Age was also positively associated with exclusive EC use (RRR = 1.14; 95% CI: 1.05–1.23) and dual use (RRR = 1.23; 95% CI: 1.13–1.35). Regarding socioeconomic class, adolescents from class B had lower odds of dual use compared to those from class A (RRR = 0.70; 95% CI: 0.55–0.90), while those from class D/E had lower odds of exclusive EC use (RRR = 0.52; 95% CI: 0.30–0.89) (Table 3).

## Discussion

This study examined psychosocial factors associated with distinct patterns of nicotine use among adolescents living in a context where the sale of electronic cigarettes has been prohibited for more than a decade. Perceived accessibility emerged as one of the strongest correlates of both exclusive e-cigarette and dual use, suggesting that adolescents’ perceptions of product availability remain closely linked to nicotine use despite the restrictive regulatory context. We also found that exclusive e-cigarette use and dual use were more frequent among girls, whereas exclusive conventional cigarette use was more common among boys. Furthermore, the strength of associations between psychosocial factors and nicotine use differed across patterns of use, with consistently stronger associations observed among dual users. In particular, perceived peer use, ease of access, and the belief that e-cigarettes help reduce conventional cigarette smoking were associated with increased likelihood of exclusive e-cigarette and dual use, whereas perceiving e-cigarettes as highly addictive was associated with lower odds of both outcomes.

Our results indicate that the prevalence of EC use, either exclusively or in combination with conventional cigarettes, was higher among girls, contrasting with the higher prevalence of exclusive conventional cigarette use among boys. These findings reflect a dynamic and evolving landscape. For instance, while Torres-Alvarez [22] reported an increase in EC use among girls in the U.S., Izquierdo-Condoy [12] found higher prevalence among boys in six Latin American countries. In Brazil, data from the 2019 PeNSE survey still indicated higher smoking rates among boys [23]. However, recent findings from the National Survey on Alcohol and Drugs already suggest a potential reversal in this trend, with greater EC use among girls [24]. This increase in female users may be related to product features such as bright colors, discreet design, and sweet flavors—characteristics often associated with female preferences [25]. It is worth noting that, historically, due to cultural norms and male-oriented marketing strategies, women have represented a minority of smokers, making this shift particularly concerning from a public health perspective [2].

Another key finding relates to normative beliefs and adolescents’ perceptions of peer behavior. The greater the perception that friends used ECs, the higher the odds of both exclusive and dual use. This is consistent with previous studies, including a review conducted in Latin American countries that highlighted the influence of peers and family as significant motivators for initiating EC use [12]. Such influence may be explained by the central role of social bonds during adolescence and the need for group belonging—an idea widely recognized in prevention science and foundational to many prevention interventions, particularly those based on social influence models [26].

Perceived ease of access also emerged as a factor associated with EC and dual use. Recent data also indicate that adolescents who use ECs perceive access as easier, while non-users tend to perceive it as more difficult [24]. These findings support existing evidence that perceived barriers to access serve as important protective factors against adolescent substance use [27]. This reinforces the importance of public policies aimed at limiting access and enforcing restrictions on the illegal sale of these devices, particularly in contexts where their sale is prohibited. Moreover, it highlights the fragility of Brazilian regulatory systems and the difficulty of ensuring effective enforcement, underscoring the urgent need for specific and efficient policies to curb EC use among youth [12].

Another notable result is the association between the perception of high addictive potential and lower odds of both EC and dual use. This supports previous studies showing that adolescents who use ECs tend to underestimate their risks and overestimate their benefits compared to non-users [28–30]. This tendency seems even more pronounced among those with more harmful use patterns, such as dual users, as prior research suggests that high-risk perception provides stronger protection against such patterns [21]. These findings reinforce the need for public policies that invest in educational strategies aimed at promoting more realistic perceptions of EC risks and addictive potential among adolescents [31].

When considering the three patterns of consumption collectively, we observed a gradient in the strength of associations between these outcomes and the perceptions and beliefs investigated. Although factors such as normative beliefs, access perception, and risk perception were associated with all patterns, the associations were consistently stronger among dual users, suggesting greater vulnerability within this subgroup [14]. Similar findings have been reported in international studies, which identify dual use as a pattern more sensitive to psychosocial and risk perception factors and, therefore, requiring specific attention in preventive strategies [32, 33]. These findings underscore the importance of treating the three usage patterns as distinct outcomes in risk and perception analyses, particularly to inform targeted nicotine prevention interventions for adolescents.

Despite the relevance of these findings, some limitations must be acknowledged. First, the cross-sectional design precludes causal inference regarding the relationships between beliefs and perceptions and patterns of nicotine use. Second, data were self-reported and thus susceptible to recall bias or social desirability bias, especially regarding sensitive behaviors such as substance use. Additionally, the sample was limited to small municipalities in a single Brazilian state, which may limit the generalizability of findings to adolescents from other regions or more complex urban contexts. Factors such as EC availability, regulatory enforcement, marketing exposure, and peer influence may vary substantially in larger and more diverse urban areas. Urbanization level has been associated with tobacco product consumption and smoking cessation: while prevalence tends to be higher in less urbanized areas, cessation is more frequent in more urbanized contexts 34. Therefore, the findings should be interpreted in light of these limitations, and any conclusions drawn must consider the specific context of the study.

In summary, this study showed that exclusive e-cigarette use and dual use are common among Brazilian adolescents living in a context where e-cigarette sales have been prohibited for more than a decade. Perceived accessibility, peer norms, and beliefs about addictiveness and smoking reduction were associated with different patterns of nicotine use, with progressively stronger associations observed from exclusive use to dual use. This gradient suggests that psychosocial factors may play an increasingly important role across nicotine use patterns, particularly among dual users. Together, these findings highlight the importance of complementing regulatory measures with prevention strategies that address psychosocial factors associated with adolescent nicotine use while reinforcing actions aimed at reducing youths’ access to e-cigarettes.
